# Hereditary intrinsic factor deficiency in China caused by a novel mutation in the intrinsic factor gene—a case report

**DOI:** 10.1186/s12881-020-01158-z

**Published:** 2020-11-10

**Authors:** Jing Ruan, Bing Han, Junling Zhuang, Miao Chen, Fangfei Chen, Yuzhou Huang, Wenzhe Zhou

**Affiliations:** Department of Hematology, Peking Union Medical College, Hospital, Chinese Academy of Medical Sciences, No.1 Shuaifuyuan, Dongcheng District, Beijing, 100730 China

**Keywords:** Megaloblastic anemia, Cobalamin deficiency, Intrinsic factor

## Abstract

**Background:**

Hereditary intrinsic factor deficiency is a rare disease characterized by cobalamin deficiency with the lack of gastric intrinsic factor because of gastric intrinsic factor (*GIF*) mutations. Patients usually present with cobalamin deficiency without gastroscopy abnormality and intrinsic factor antibodies.

**Case presentation:**

A Chinese patient presented with recurrent severe anemia since age 2 with low cobalamin level and a mild elevation of indirect bilirubin. The hemoglobin level normalized each time after intramuscular vitamin B12 injection. Gene test verified a c.776delA frame shift mutation in exon 6 combined with c.585C > A nonsense early termination mutation in exon 5 of *GIF* which result in the dysfunction of gastric intrinsic factor protein. The hereditary intrinsic factor deficiency in literature was further reviewed and the ancestry of different mutation sites were discussed.

**Conclusions:**

A novel compound heterozygous mutation of *GIF* in a Chinese patient of hereditary intrinsic factor deficiency was reported. It was the first identified mutation of *GIF* in East-Asia and may indicate a new ancestry.

## Background

Vitamin B12 or cobalamin deficiency is characterized by megaloblastic anemia with neurological problems and can be caused by numerous acquired and inherited diseases [1]. Decreased intake, impaired gastric absorption including pernicious anemia and gastrectomy, impaired intestinal absorption caused by parasites infection are common acquired causes. As for inherited diseases, the mechanisms vary from impaired cobalamin absorption, defects of cobalamin transport to failure of cellular cobalamin metabolism. Two hereditary diseases have been found to cause cobalamin malabsorption including Imerslund-Grasbeck syndrome (IGS) and hereditary intrinsic factor deficiency (IFD) [2].

IFD is caused by homozygous or compound heterozygous mutation in the gene of gastric intrinsic factor on chromosome 11q12. It presents in early childhood with the lack of gastric intrinsic factor, while the gastric acid secretion is normal and no autoantibody to intrinsic factor is found. It is a rare disease mostly occurring in the Europe [3]. Here we present a Chinese family identified to have hereditary intrinsic factor deficiency with a new mutation site in *GIF* that has not been reported.

## Case presentation

### Clinical manifestation

**Fig. 1:**
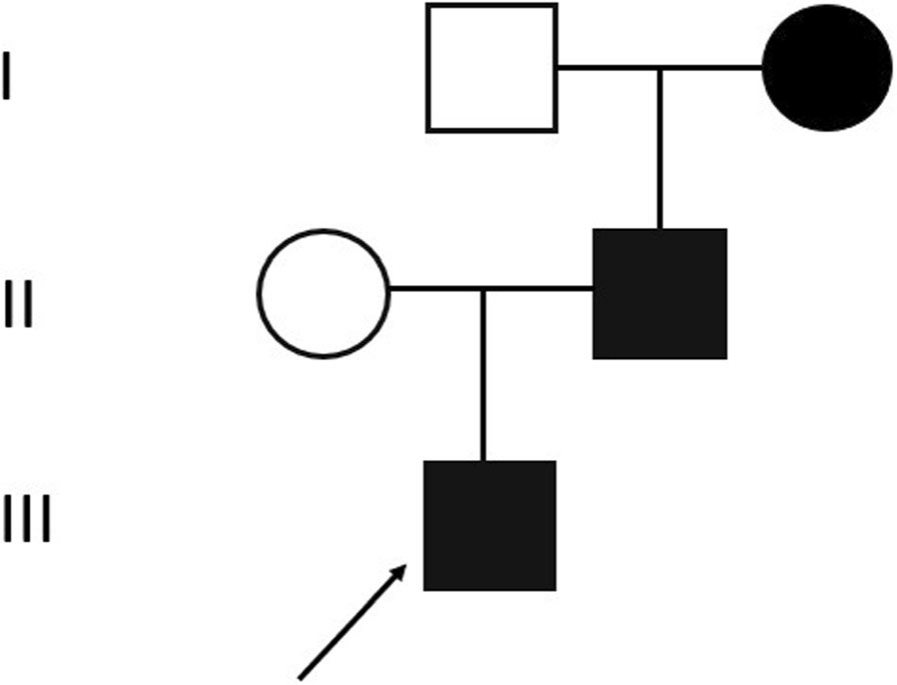
Family tree of the proband

The proband was a 16-year-old Chinese boy with a history of patent ductus arteriosus. The child was initially evaluated for severe anemia at the age of 2. Severe megaloblastic anemia, low cobalamin level and a mild elevation of indirect bilirubin were found. The hemoglobin level normalized after intramuscular vitamin B12 injection and oral folate with unknown dosage. He did not have severe symptoms the following years. At age 8, he was admitted to the hospital for the recurrence of anemia induced by upper airway infection. He also had jaundice and tea-colored urine. The hemoglobin was 57 g/L, mean cell volume was 97.8fL, and the cobalamin level was 80 pg/mL. Hemolytic anemia was also found with the indirect bilirubin to be 43.0umol/L and lactate dehydrogenase to be 1832U/L. Rous test, Coombs test, erythrocyte osmotic fragility test, glucose-6-phosphate dehydrogenase activity and the count of CD55/CD59 negative cells were normal. The ultrasound of the spleen showed a mild enlargement. Bone marrow indicated megaloblastic erythroid hyperplasia. He was treated with intramuscular injection of vitamin B12 at the dosage of 0.5 mg every other day and his hemoglobin increased to 114 g/L. The patient had recurrent anemia 4 times from age 10 to 16 and the hemoglobin regained normal after B12 supplement. He came to our hospital for further examination. We found the antibody of intrinsic factor was negative and his gastroscope was normal. Since his grandmother and his father also had mild anemia, cobalamin concentration was then tested, and they were both diagnosed to have vitamin B12 deficiency. (Fig. 1). Therefore, hereditary disease was further suspected, and we performed genome sequencing to convince it.

### Analysis of genome sequencing

**Fig. 2:**
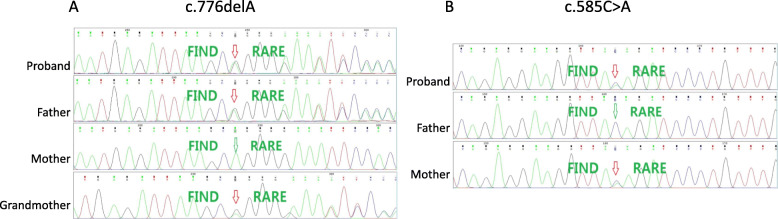
Sanger validation of the compound heterozygous mutation in *GIF* gene

Genomic DNA was isolated from the peripheral blood of the patient. SeqCap EZ Choice XL Library (Roche NimbleGen) was used to hybridize the exons and adjacent intron regions (50 bp) of 238 genes related to hereditary hematological diseases. After amplification and purification, high-throughput sequencing was performed by Illumina. Analysis was performed using the hg19 annotation information provided by UCSC. Genomic DNA samples of the patient’s parents and grandparents were then isolated for validation of the identified mutations by Sanger sequencing. The patient had compound heterozygous mutation in *GIF* gene (Fig. 2). There was a c.776delA mutation combined with c.585C > A mutation on the other allele. c.776delA (p.Q259Rfs*17) is a frame shift mutation in exon 6 caused by 1 bp deletion which would result in abnormal protein translation. It was also found in father and grandmother of the proband. c.585C > A (p.Y195X) is a nonsense mutation in exon 5 that would lead to the early termination of the gene coding protein and was also detected in his mother. Since there are reports for mutations of the downstream coding sites that could result in abnormal function of the gastric intrinsic factor, these two mutations are both thought to cause pathological changes.

## Discussion and conclusions

Juvenile cobalamin deficiency usually presents with various hematological problems ranging from mild weakness to lift-threatening anemia. Patients may present with yellow skin caused by combined anemia and jaundice. Rate of infection is also increased due to neutropenia, thrombocytopenia and megaloblastic anemia. The neurologic abnormalities are also variable and may not be recognized without attention. Growth retardation and learning difficulties are more commonly seen in the juveniles [4]. Patients may also have dementia, psychological problems and neurodegeneration of the spinal cord.

Although inborn cobalamin deficiency is rare, with social and economic development, dietary and infectious causes of cobalamin deficiency are decreased and the prevalence of inherited cobalamin deficiency has increased [5]. Inborn errors affecting intestinal cobalamin absorption, transport of cobalamin in blood, uptake of cobalamin by cells or intracellular cobalamin metabolism could all cause cobalamin deficiency. As for hereditary cobalamin malabsorption, IGS caused by *CUBN* or *AMN* mutations and IFD caused by *GIF* mutations are most common ones. Schilling test was used in the past to differentiate IFD from IGS and has been obsoleted because of invasiveness and lack of access to the radiolabeled vitamin B12. Genetic testing is now available for the validation and classification of the inherited cobalamin malabsorption. Tanner et al. 2012 [3] observed 22%, 42%, 36% of the mutations in *GIF*, *CUBN* and *AMN* genes respectively in a large genetic screening study of 154 families or patients with hereditary deficiency of vitamin B12 absorption.

Hereditary intrinsic factor deficiency is characterized by cobalamin deficiency with the lack of gastric intrinsic factor because of *GIF* mutations. The gastric acid secretion and gastroscopy are often normal and no autoantibodies to intrinsic factor should be found. We summarized the mutations in *GIF* from previous literature in Table 1. The first report with identified genetic mutations was in 2004 [6]. Yassin et al. [6] identified a 4-base deletion (c183_186delGAAT, p.Met61fs) in exon 2 in an 11-year-old girl with severe anemia and cobalamin deficiency. This homozygous mutation seems to originate in Africa and was also reported by Tanner et al. 2005 [7] and Ament et al. 2009 [8]. In Exon 2, another 2 homozygous mutation sites were also found including the c.137C > T (p.Ser46Leu) and c.161delA (p.Asn54fs). There are also homozygous mutations in exon 5 (c.685G > A) [3], exon 8 (c.1175_1176insT) [7] and exon 9 (c.1222G > A) [9]. Intron mutations in the splice site can also cause abnormal protein structures and functions. The most numerous one was c.79 + 1G > A mutation commonly found in Europe. Not only the homozygous mutation of this site but also in combination with other defects including a 3-terminal deletion in intron 8 and 3 different missense mutation sites (c.137C > T, c.290 T > C [10], c.673A > C). c.80-1G > A in intron 1 [7] and c.1073 + 5G > A [11] homozygous mutations were reported respectively in West Asia area. There are also compound heterozygous mutations in two exons that generate IFD [8, 12]. Notably, Chery et al. [13] reported 2 IFD family who carried *FUT2* rs601338 secretor variants that impairs GIF secretion. This variant in combination with *GIF* heterozygous mutation worsened the B12 status.

**Table1:** Summary mutations in the GIF gene according to previous literature (add to page 5 line 19)

DNA mutation	Region	Genotype	Predicted consequence	References	Origin
c.79 + 1G > A	intron 1	hom	splice site mutation	Tanner et al. 2005 [5], Tanner et al. 2012 [3]	France, Norway, USA
c.79 + 1G > A & del Intron 8 to distal of 3′-end	intron 1 & del	comp het	splice site mutation & partial gene deletion	Tanner et al. 2012 [3]	Norway
c.79 + 1G > A & c.137C > T	intron 1 & exon 2	comp het	splice site mutation & p.Ser46Leu	Tanner et al. 2012 [3]	USA (Western Europe?)
c.79 + 1G > A & c.290 T > C	intron 1 & exon 3	comp het	splice site mutation & p.Met97Thr	Overgaard et al. 2010 [10]	USA (Western Europe?)
c.79 + 1G > A & c.673A > C	intron 1 & exon 5	comp het	splice site mutation & p.Ser225Arg	Tanner et al. 2012 [3]	Siberia
c.80-1G > A	intron 1	hom	splice site mutation	Tanner et al. 2005 [5]	Kuwaiti
c.137C > T	exon 2	hom	p.Ser46Leu	Tanner et al. 2005 [5], Tanner et al. 2012 [3]	Turkey
c.161delA	exon 2	hom	p.Asn54fs	Tanner et al. 2005 [5]	Turkey
c.183_186delGAAT	exon 2	hom	p.Met61fs	Yassin et al. 2004 [6], Tanner et al. 2005 [5], Ament et al. 2009 [8]	UK (Jamaican), African American
c.183_186delGAAT & c.659 T > C	exon 2 & exon 5	comp het	p.Met61fs & p.Ile220Thr	Ament et al. 2009 [8]	USA (African, European)
c.256 + 2 T > G & c.659 T > C	intron 2 & exon 5	comp het	splice site mutation & p.Ile220Thr	García Jiménez et al. 2008 [12]	Spain
c.290 T > C & ?	exon 3 & ?	comp het	p.Met97Thr & ?	Tanner et al. 2012 [3]	Finland
GIF c.290 T > C & FUT2 rs601338 461GG variant		comp het	GIF p.Met97Thr & FUT2 secretor variant	Chery et al. 2013 [13]	France
c.431_438delAGAAGAAC & c.974_975insG	exon 4 & exon7	comp het	p.Gln144fs & p.Val325fs	Tanner et al. 2012 [3]	Austria
c.435_437delGAA & FUT2 rs601338 461GG variant		comp het	p.Lys145_Asn146delinsAsn & FUT2 secretor variant	Chery et al. 2013 [13]	France
c.469 T > C & ?	exon 4 & ?	comp het	p.Phe157Leu & ?	Tanner et al. 2012 [3]	USA (Lebanese)
c.685G > A	exon 5	hom	p.Ala229Thr	Tanner et al. 2012 [3]	Turkey, Germany (Lebanese)
c.938C > T & ?	exon 7 & ?	comp het	p.Thr313Ile & ?	Tanner et al. 2012 [3]	Israel (Arabic)
c.1073 + 5G > A	intron 7	hom	splice site mutation	Sturm et al. 2013 [11]	USA (Chaldean)
c.1175_1176insT	exon 8	hom	p.Thr393fs	Tanner et al. 2005 [5]	Turkey
c.1222G > A	exon 9	hom	p.Glu408Lys	Lund Leunbach et al. 2011	Danish

As for this patient, we found a c.776delA frame shift mutation in exon 6 combined with c.585C > A nonsense early termination mutation in exon 5 and the two mutation alleles were inherited from his parents respectively. This compound heterozygous mutation caused the severe loss of function of the encoding gastric intrinsic factor protein and resulted in cobalamin deficiency. It was the first identified novel mutation of *GIF* in East-Asia and may indicate a new ancestry.

The treatment is merely vitamin B12 administration by several routes including intramuscular and oral. Our patient recovered the hemoglobin level each time after applying vitamin B12. In fact, treatment with life-long cobalamin administration at regular intervals is life-saving and prevents further deterioration [14]. Early diagnosis and detection by genetic methods in such juvenile cases followed by regular treatment may help avoid severe hematological, neurological and developmental problems.

In summary, we identified a case diagnosed to be hereditary intrinsic factor deficiency firstly with genetic information in the East Asia. A novel compound heterozygous mutation of *GIF* was reported which may indicate a different origin from previous literature.

## Data Availability

The datasets generated and/or analyzed during the current study are available in the NCBI BioProject database under the accession number PRJNA669950. The hg19 annotation dataset could be reached at https://genome.ucsc.edu/.

## Abbreviations

GIF: Gastric intrinsic factor
IGS: Imerslund-Grasbeck syndrome
IFD: Intrinsic factor deficiency
